# Biomarkers for prostate cancer: present challenges and future opportunities

**DOI:** 10.4155/fso.15.72

**Published:** 2015-12-17

**Authors:** Pranav Sharma, Kamran Zargar-Shoshtari, Julio M Pow-Sang

**Affiliations:** 1 Department of Genitourinary Oncology, Moffitt Cancer Center, 12902 Magnolia Drive, Office 12538, Tampa, FL 33612, USA

**Keywords:** biomarkers, circulating tumor cells, diagnosis, genetic panels, prognosis, prostate cancer

## Abstract

Prostate cancer (PCa) has variable biological potential with multiple treatment options. A more personalized approach, therefore, is needed to better define men at higher risk of developing PCa, discriminate indolent from aggressive disease and improve risk stratification after treatment by predicting the likelihood of progression. This may improve clinical decision-making regarding management, improve selection for active surveillance protocols and minimize morbidity from treatment. Discovery of new biomarkers associated with prostate carcinogenesis present an opportunity to provide patients with novel genetic signatures to better understand their risk of developing PCa and help forecast their clinical course. In this review, we examine the current literature evaluating biomarkers in PCa. We also address current limitations and present several ideas for future studies.

## Abstract (lay abstract)

**Lay abstract:** Several biological markers as well as collections of genetic mutations have been identified by researchers that are associated with the development and aggressiveness of prostate cancer. They can also predict death from prostate cancer in combination with other known risk factors. Some are commercially available for use in current clinical practice. These personalized genetic profiles can help select patients that are most likely to benefit from definitive treatment with either surgery or radiation therapy. Future studies, however, need to focus on biological manipulation of known high-risk genes to reduce the incidence of this disease for future generations.

**Figure 1.**
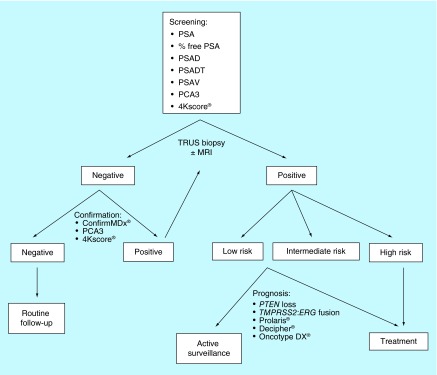
**Proposed algorithm for prostate cancer management.** MRI: Magnetic resonance imaging; PCA3: Prostate cancer antigen 3; PSA: Prostate specific antigen; PSAD: PSA density; PSADT: PSA doubling time; PSAV: PSA velocity; TRUS: Transrectal ultrasound.

## Background

Prostate cancer (PCa) remains the most common urologic malignancy and the second most common cause of cancer death for men in developed countries [1]. Since the advent of early detection efforts such as PSA screening and increased public awareness, PCa is frequently diagnosed in its initial stages. Reports over the past decade also indicate that a significant percentage of men with newly diagnosed PCa may be managed by active surveillance (AS) [2]. PCa screening, however, is still controversial since early detection can lead to the diagnosis and overtreatment of clinically insignificant disease with long-term effects on patient quality of life [3,4]. Additionally, no reliable screening tool exists that can consistently differentiate indolent versus potentially life-threatening disease [5].

A personalized, patient-centered approach to both the diagnosis and treatment of PCa is necessary due to the variability in disease behavior as well as the multitude of treatment options. Additionally, an individualized approach may improve prognostic criteria and minimize the risk of unnecessary treatment and any associated impairment in quality of life.

Improvements in technology aimed at genetic analysis have led to the discovery of an abundance of new biomarkers that may be utilized in the prediction of PCa incidence, outcomes and response to therapy [6]. The characterization of genetic mutations in tumor tissue through advanced technologies, such as microarray analyses and next-generation sequencing, can subsequently create personalized road maps to guide clinical decision-making due to a better understanding of a patient's risk of progression [7].

We have summarized below the utilization of PCa biomarkers in current clinical practice, their limitations and possible future considerations for their use. We discuss the potential application of biomarkers as a screening or diagnostic tool in men at high risk of developing PCa and as a prognostic tool to discriminate indolent versus aggressive disease, aiming to improve treatment strategies in PCa patients at either a localized or advanced stage. Metabolomic PCa biomarkers, which have recently come to the forefront as a noninvasive investigational tool for PCa detection using *in vivo* magnetic resonance spectroscopy (MRS), are not discussed in the following review as they have yet to develop mainstream utilization in current clinical practice [8,9]. There is a growing body of evidence, however, that supports the inclusion of MRS in PCa diagnosis and treatment due to their improved sensitivity and specificity in predicting disease prognosis and for patient stratification through the metabolic fingerprint of different biofluids [10–14].

## Genetic risk factors in PCa

Family history of PCa in first-generation male relatives is a well-recognized risk factor for developing the disease with twin studies suggesting that greater than 40% of PCa risk is inheritable [15]. Through the Human Genome Project, analysis of constitutional DNA has shown over 60 PCa susceptibility loci explaining approximately a third of the familial risk have been identified, and multiple single nucleotide polymorphisms (SNPs) have been correlated with risk of developing disease [16,17]. Individuals carrying five of these risk alleles were almost tenfold more likely to develop PCa than men carrying no risk alleles (odds ratio [OR]: 9.46), but inclusion of this genotype did not add significantly in the prediction of PCa when added to established risk factors such as age and family history [18].

Rarer variants may be associated with higher relative PCa risk [19]. Coding variants in the homeobox B13 (HOXB13) were found in <0.1% of controls, but 1.4% of patients with a strong family history of early-onset PCa [20]. Although PCa risk stratification including genetic variants are currently limited since they do not add appreciably to the utility of more established clinical risk factors, inclusion of higher-risk variants may improve the performance of these models.

Germline *BRCA* mutations, which have dramatic genetic implications in breast and ovarian cancer, have also been associated with PCa. *BRCA1* and *BRCA2* play central roles in DNA repair by homologous recombination, which is the mechanism that cells use to repair double-stranded breaks induced, for example, by platinum-based chemotherapeutic agents or ionizing radiation (XRT). Previous studies have demonstrated that *BRCA1* and *BRCA2* mutations are predictive factors for response to platinum-based chemotherapy in breast and ovarian cancer [21,22]. *BRCA2* mutations have subsequently been shown to confer a higher risk of PCa in men ≤65 years of age (OR: 8.6) [23], and they were an independent prognostic factor for disease-specific survival (DSS) in all stages of PCa including localized disease [24]. Castro *et al*. evaluated tumor features and outcomes of 1302 patients with local or locally advanced PCa (including 67 *BRCA* mutation carriers) and found that at 3, 5 and 10 years after treatment (radical prostatectomy [RP] or XRT), noncarriers were more likely to be free from metastasis than carriers (97 vs 90%, 94 vs 72% and 84 vs 50%, respectively, [p < 0.001]). Noncarriers also had better 3-, 5- and 10-year cancer-specific survival (CSS) rates than carriers (99 vs 96%, 97 vs 76% and 85 vs 61%, respectively, [p < 0.001]) [25].

Genetic characterization may play an important clinical role in the future. HOXB13, for example, is associated with low-risk PCa, while *BRCA2* is associated with intraductal carcinoma, an early-onset aggressive PCa with a poor prognosis [26]. These susceptibility genes are also racially and ethnicity dependent and act in an additive fashion. Genotyping all men at susceptibility loci, however, could also lead to the overdiagnosis and overtreatment of potentially indolent PCa, subjecting more patients to the morbidity associated with treatment. It is our hope that screening and preventative therapy could be tailored according to risk stratification algorithms incorporating genetic mapping along with family history [27].

## Biomarkers in PCa diagnosis

### Prostate-specific antigen & its derivatives

The most common current screening test for PCa is a measurement of the serum concentration of prostate-specific antigen (PSA), which currently cannot reliably predict adverse pathological features [28]. Additionally, there is no single cutoff value for PSA that can accurately distinguish patients with PCa from those without [29]. Age-specific upper reference limits for serum total PSA have been created and adjusted according to race (i.e., Caucasian, African–American, etc.) [30,31], and percent-free PSA (free-to-total PSA ratio) may also improve the performance of PSA-based testing [32]. SNPs at six loci have additionally been identified that indicate a significant association between PSA levels and patients without PCa, suggesting that PSA thresholds for biopsy could be personalized based on the patient's genotype at these loci [33]. A brief description of the various PSA-based tests frequently utilized in clinical practice is listed in Table 1, and a summary of commercially available biomarkers commonly used in clinical practice for PCa detection is listed in Table 2.

### PSA kinetics (velocity & doubling time)

There has been considerable uncertainty about PSA kinetics for decisions about prostate biopsy for diagnosis. Recent studies, including analyses of cohorts from all the major randomized trials of localized PCa, have failed to find any evidence that PSA velocity (PSAV) and application of PSA cut points are of benefit in screening. Additionally, PSA dynamics are related to cancer growth rates only partially due to both malignant and nonmalignant processes contributing to PSA fluctuations. Nevertheless, PSA dynamics have been claimed to aid in the long-term prediction of PCa detection [38], and guidelines for PCa diagnosis include a recommendation that men with a PSAV greater than 0.35 ng/ml per year should consider biopsy even if their total PSA is low [39]. Others, however, have refuted the concept that the rate of change of PSA is of any value [40]. A brief definition of PSA kinetics is provided in Table 1.

Ulmert *et al*. tested whether PSAV improved the accuracy of a model using PSA level to predict long-term risk of PCa diagnosis in 4907 screened men (of which 443 [9%] were diagnosed with PCa) [41]. The authors found that PSAV correlated strongly with PSA level (r = 0.93) and that adding PSAV did not significantly increase the accuracy of PSA to predict PCa development. These results were also unchanged, if the analysis was restricted to patient with advanced cancer at diagnosis.

In a systemic review of PSA kinetics in the diagnosis of PCa, PSAV added little predictive value to PSA alone (area under curve [AUC] = 0.83 vs 0.81) [42]. Several other groups have reported that PSAV and PSA doubling time (PSADT) failed to improve the specificity of PSA for biopsy [43,44]. PSAV was not shown to aid in PCa detection in men with prior negative biopsies [45]. Data from the REDUCE trial also clearly indicate that changes in PSAV are unable to predict the risk of a repeat positive biopsy after initially negative findings [46]. In a study from the PCPT, PSAV added very little to the AUC of standard predictors alone (0.709 vs 0.702) with no improvement in the detection of high-grade cancer (0.792 reduced to 0.791) [47]. Using PSAV did not improve the sensitivity of PSA and would lead to many additional biopsies per year without a corresponding increase in the number of high-grade cancers detected.

Conversely, there are several studies that advocate the use of PSAV and PSADT in addition to total PSA for the detection of PCa. In a retrospective cohort of 219,388 men with at least three PSA measurements, Wallner *et al*. reported that the annual percent change in PSA accurately predicted the presence of PCa (AUC = 0.963) and the presence of aggressive disease (AUC = 0.955) with more accuracy than a single measurement of PSA alone (AUC = 0.727) [34]. Orsted *et al*. also showed that when long-term PSAV was added to models already including the baseline PSA value, the age-adjusted hazard ratio (HR) for PCa detection and PCa-related death increased from 2.7 to 5.3 and from 2.3 to 3.4, respectively [48]. The authors, therefore, concluded that long-term PSAV in addition to baseline PSA values improved the classification of PCa risk and mortality.

Due to the conflicting evidence with regards to PSAV and PSADT as a screening tool for PCa diagnosis, several expert consensus recommendations have been developed [49]. These include: first, high PSAV is not an indication for biopsy; second, men with a low PSA but a high PSAV should have another PSA drawn at a shorter time interval; third, men with an indication for biopsy should be biopsied regardless of PSAV; fourth, changes in PSA after negative biopsy findings do not indicate the need for repeat biopsy, and lastly, PSA monitoring over time can aid in clinical decision-making about biopsy but judgment should ultimately be informed by the clinical context.

### PCA3

PCA3 is a noncoding RNA with expression confined to the prostate, which is highly overexpressed in 95% of PCa cases compared with benign prostatic tissue [50]. Some men, however, may have a very high PCA3, but no evidence of malignancy [51]. It is commercially available as a diagnostic test that quantitatively detects PCA3 RNA expression in the urine and prostatic fluid after prostatic massage with a score higher than 35 in the urine correlating with an average sensitivity and specificity of 66 and 76%, respectively, for the diagnosis of PCa (compared with a sensitivity of 65% and specificity of 47% for PSA alone) [35]. Elevated PCA3 can also increase the probability of a positive repeat prostate biopsy in men with one or two prior negative biopsies, and it is the US FDA approved for this indication [52]. As such, it may play a complimentary role to PSA alone in PCa screening.

### Prostate health index

The Beckman Coulter Prostate Health Index (PHI) combines total, free and [-2]proPSA into a single score to improve PCa detection [53]. As a component of free PSA, [-2]proPSA is more specific for PCa than total or free PSA alone [36,54]. Catalona *et al*. evaluated the PHI in a prospective, multi-institutional trial of 892 men with a PSA between 2 and 10 ng/ml and a negative digital rectal examination (DRE) [55]. The predictive ability of PHI to diagnose PCa (AUC = 0.703) exceeded that of serum PSA (AUC = 0.525), free PSA (AUC = 0.615), p2PSA (the primary form in PCa tissue) (AUC = 0.557) or percent-free PSA (AUC = 0.648). PHI also performed better than percent-free PSA in its ability to discriminate PCa with a Gleason score (GS) ≥4 + 3 = 7 versus lower grade PCa or benign histology (AUC = 0.724 vs 0.670).

Loeb *et al*. also externally validated the PHI in a multicenter prospective trial of 658 men aged ≥50 years with a PSA of 4–10 ng/ml and normal DRE who underwent prostate biopsy [56]. Based on Epstein criteria, the PHI was able to detect clinically significant disease (AUC = 0.698) with more accuracy than percent-free PSA (AUC = 0.654), [-2]proPSA (AUC = 0.550) or PSA (AUC = 0.549) alone, suggesting its use to reduce prostate biopsies and the overdiagnosis of indolent disease.

### Kallikrein panel

Another promising serum-based biomarker is the kallikrein panel (4k-panel) (OPKO Health Inc., FL, USA) that consists of total PSA, free PSA, intact PSA and human kallikrein-related peptidase 2 (KLK2) [57]. This prostate ‘4Kscore^®^’ was shown by Vickers *et al*. to improve the predictive accuracy of PCa detection over PSA alone (AUC: 0.711 vs 0.585) with a reduction in the biopsy rate by 362 for every 1000 men with elevated PSA [58]. Similar results have been seen in externally validated populations in France and The Netherlands with an improvement in the detection of high-grade cancers (AUC increased from 0.77 to 0.87 and 0.76 to 0.87, respectively) [59,60]. When comparing the 4k-panel to the PHI, both similarly improved discrimination when predicting both PCa and high-grade PCa, reducing the number of unnecessary biopsies compared with PSA alone [61]. The 4k-panel has also been shown to enhance the prediction of lethal PCa associated with metastasis compared with PSA alone, providing an additional use as a screening tool for men with elevated PSA [62]. In patients with elevated PSA (>3 ng/ml), low percent-free PSA or suspicious DRE, the 4k-panel of kallikrein markers improved discrimination over age and PSA for high-grade cancer (GS ≥7; AUC = 0.77 vs 0.720; p = 0.002), reducing the number of biopsies by 236 per 1000 to detect 195 of 208 high-grade cancers [63].

### *TMPRSS2*: *ERG* fusion & β-microseminoprotein

Tomlins *et al*. recognized one of the most frequent gene-specific alterations in PCa represented by a fusion between the*TMPRSS2* gene and the v-ets avian erythroblastosis virus *ERG* gene [64]. Detection of the *TMPRSS2*: *ERG* fusion in the urine after prostatic massage has been reported to yield more than 90% specificity and 94% positive predictive value (PPV) for PCa detection [65]. The sensitivity of this assay, however, is low since 40–50% of prostate tumors carry this fusion, but it can be improved in combination with PCA3. Urinary detection of PCA3 and *TMPRSS2*: *ERG* with serum PSA levels has also been reported to improve PCa screening performance compared with PSA alone [66]. Other biomarkers in an active area of research include urinary concentrations of β-microseminoprotein, whose levels are decreased in PCa compared with normal prostate tissue [67]. Decreased urinary levels of β-microseminoprotein have been shown to improve PCa diagnosis over urinary PSA but not serum PSA, so further testing is required to demonstrate any potential utility of this test.

### DNA methylation markers

Hypermethylation of CpG islands in the promoter regions of cancer-associated genes is linked to PCa [68]. Three of these genes include *GSTP1* [68], which is involved in DNA detoxification; *APC*, which is involved in cell apoptosis, migration and adhesions [69]; and *RSSF1*, which is involved in cell cycle regulation [70]. A recent meta-analysis concluded that GSTP1 methylation occurs in up to 90% of PCa cases (on both tissue biopsy and RP specimens), while it is only seen in 5% of noncancerous controls [37]. *GSTP1* is also the most widely reported hypermethylated gene in PCa [71]. Since DNA hypermethylation of key genes occurs early during oncogenesis, it is ideally suited for PCa detection [72]. Due to the concept of epigenetic field effect in prostate carcinogenesis [73], investigators have evaluated whether biopsy samples taken with negative pathology findings may produce a positive molecular result since genetic alterations occur in histopathologically nonmalignant tissue that is contiguous with cancerous tissue [74].

In the MATLOC study, Stewart *et al*. determined the degree of methylation of *GSTP1, APC* and *RASSF1* to detect PCa in initial histopathologically negative biopsy samples from men who were subsequently rebiopsied [75]. This epigenetic assay resulted in a negative predictive value (NPV) of 90% (compared with 70% for histopathology alone), and it was an independent predictor of PCa detection up to 30 months before repeat biopsy. In the follow-up DOCUMENT study, this epigenetic test was externally validated in 350 subjects with an initial negative prostate biopsy across five urological centers in the USA [76]. It resulted in a NPV of 88% and again was an independent predictor of PCa detection on multivariate analysis after correcting for age, PSA, DRE, histopathological characteristics on first biopsy and race. Negative findings of this assay, therefore, could be used to decrease concern about unsampled cancer and avoid unnecessary repeat prostate biopsies. The impact of this epigenetic test (commercially available as ConfirmMDx^®^ [MDxHealth, CA, USA]) on rebiopsy rates was recently surveyed at five centers, and among 138 patients with a negative assay, only six patients (4%) underwent repeat biopsy [77].

## Biomarkers in PCa prognosis

### PSA kinetics & PSA density

The role of PSA kinetics (PSAV and PSADT) as a tool for PCa prognosis has been extensively studied and is still highly ambiguous as a predictive factor in disease aggressiveness [78]. Khatami *et al*. and Soloway *et al*. both reported that preoperative PSADT was a statistically significant predictor of disease progression and PSA relapse after RP [79,80]. Ross *et al*., however, reported a 35% rate of disease progression on repeat biopsy in an AS program at median follow-up of 2.9 years, and neither PSAV nor PSADT was a significant predictor of progression on univariate analysis [28]. Whitson *et al*. similarly revealed that PSADT was not significantly associated with risk of biopsy disease progression (grade or volume increase) [81]. Iremashvili *et al*. reported that PSAV significantly predicted tumor progression in certain subgroups, including men undergoing their fourth or later surveillance biopsy, but in the overall population, there was no significant increase in the predictive accuracy compared with PSA alone [82]. Additionally, PSADT was not associated with biopsy progression in any group in this study. Conversely, San Francisco *et al*. found that PSAV along with PSADT and family history was highly predictive of disease progression (≥3 positive cores, GS ≥7 or >50% core volume) [83]. Finally, Patel *et al*. examined PSAV risk count (number of times PSAV exceeded 0.4 ng/ml per year), which outperformed standard PSAV as a predictor of disease progression on AS [84]. The 5-year probability of biopsy reclassification (GS >6, ≥3 positive cores, >50% core volume) was 9.7, 18.7 and 39.5%, respectively, with risk counts of 0, 1 and 2 (p < 0.01), respectively, and men with a risk count higher than 1 had a four-times greater risk of reclassification on multivariate analysis. For men with a risk count of 0–1, the NPV for reclassification the following year was 91%, providing a potential means to reduce invasive biopsies. Use of PSA kinetics as a stand-alone test to determine PCa progression, such as in AS programs, yields highly mixed results, but it could be used to trigger further diagnostic intervention such as MRI or saturation biopsy.

PSA density (PSAD), on the other hand, may predict GS upgrading in AS protocols for low-risk disease [85]. Barayan *et al*. and Dall'Era *et al*. both reported that a PSAD >0.15 ng/ml per cubic centimeter at diagnosis was an important predictor for disease progression and increasing GS on repeat biopsy [86,87]. Venkitaraman *et al*. also showed that PSAD was a predictor of histologic disease progression (primary GS ≥4, >50% positive cores or GS increase from ≤6 to ≥7) on univariate analysis, but it did not reach statistical significance on multivariate analysis (p = 0.069) [88]. A limitation of PSAD, however, is inaccuracy of assessing prostate volume on transrectal ultrasound [89], and loss of significance in the face of new PSA-based tests such as proPSA and PSAV risk count [90]. Tosoian *et al*. found percent [-2]proPSA and PHI provided the greatest predictive accuracy (over PSAD) for reclassification to high-grade cancer in patients on AS [91], and Hirama *et al*. showed baseline [-2]proPSA and PHI (but not PSAD) predicted pathological reclassification at 1 year in patients with low-risk disease [92].

Current recommendations suggest making treatment decisions such as AS versus surgery versus XRT or adjuvant therapy, irrespective of PSAV, PSADT or PSAD, using established prediction models based on disease stage, grade and total PSA. PSAV, however, at the time of recurrence should be entered into prognostic models to aid patient counseling since PSA changes after treatment for advanced disease can help indicate therapeutic effectiveness with a rising PSA on treatment indicating likely nonresponse.

### PCA3 & *TMPRSS2*: *ERG* fusion

The data on PCA3 and PCa prognosis are limited. Tosoian *et al*. examined urinary PCA3 in men with very low-risk PCa prospectively in an AS cohort and found that PCA3 had poor discrimination (AUC = 0.589) and was not significantly associated with short-term biopsy progression on multivariate analysis after accounting for age and diagnosis date (p = 0.15) [93]. Newer reports, however, have found a significant association between elevated PCA3 and GS ≥7 on subsequent prostate biopsy (p = 0.02) as well as an improved overall detection of PCa (82.1% sensitivity and 79.3% specificity) [94].

Although gene fusions, specifically E26 transformation-specific (ETS) fusions such as the *TMPRSS2*: *ERG* translocation, have been associated with the early onset of PCa, its clinical utility as a prognostic tool is still unclear. Nam *et al*. reported that the expression of the *TMPRSS2*: *ERG* gene fusion on polymerase chain reaction (PCR) of prostate specimens was independently predictive of biochemical recurrence (BCR) after RP (HR: 8.6) [95] as well as disease progression in a small, selected cohort of intermediate-risk patients with GS 7 adenocarcinoma [96]. In contrast, Saramaki *et al*. demonstrated lower BCR risk after RP associated with the *TMPRSS2*: *ERG* gene fusion based on fluorescence *in situ* hybridization (FISH) [97]. Steurer *et al*. found that the *TMPRSS2*: *ERG* gene fusion was associated with low-grade tumors in younger patients but not with overall outcomes [98], and Dal Pra *et al*. demonstrated no association between the *TMPRSS2*: *ERG* gene fusion and BCR in almost 250 men treated with intensity-modulated radiation therapy (IMRT) [99]. Gopalan *et al*., Hoogland *et al*. and Minner *et al*. similarly found no association between the *TMPRSS2:ERG* gene fusion and BCR or local recurrence after RP [100–102]. A large meta-analysis of 5074 men following RP also found no significant association between the *TMPRSS2*: *ERG* gene fusion and BCR or disease progression [103]. Further analysis beyond the overall expression of the gene fusion, however, can provide more significant prognostic value. FitzGerald *et al*. did not observe an association between the *TMPRSS2*: *ERG* gene fusion and overall clinical outcomes in PCa patients, but men with increased copy numbers showed poorer survival [104]. Additionally, Boormans *et al*. reported that the *TMPRSS2*: *ERG* Exon 0 gene fusion was associated with a lower risk of BCR compared with the Exon 1 fusion [105].

Presence of the *TMPRSS2*: *ERG* gene fusion may play more of a role in predicting outcomes in AS populations or watchful waiting (WW) cohorts undergoing palliative transurethral resection of the prostate (TURP). Berg *et al*. evaluated 265 men on AS and reported that expression of *TMPRSS2*: *ERG* gene fusion in biopsy specimens was independently associated with progression risk (58.6 vs 21.7%; HR: 2.45) [106]. Lin *et al*. found that on univariate analysis, urinary PCA3 and *TMPRSS2*: *ERG* expression were significantly associated with higher volume disease and higher grade disease, but this was not significant with biopsy reclassification on multivariate analysis [107].

Attard *et al*. and Hagglof *et al*. both showed that the *TMPRSS2*: *ERG* gene fusion was independently predictive of CSS and overall survival (OS) based on both FISH and immunohistochemistry (IHC) in metastatic patients undergoing palliative TURP [108,109], and this was similarly reproduced by Demichelis *et al*. and Qi *et al*. [110,111]. It was thought, therefore, that *TMPRSS2*: *ERG* expression could be related to response to androgen deprivation therapy (ADT). Boormans *et al*., however, found no association between the *TMPRSS2*: *ERG* gene fusion with ADT response in PCa patients with lymph node metastases [112]. Similarly, Leinonen *et al*. found no association between the *TMPRSS2*: *ERG* gene fusion and disease progression in ADT-treated patients [113].

Although the *TMPRSS2*: *ERG* gene fusion alone has little prognostic impact, its predictive value improves in combination with other investigative markers including other gene-expression panels [114–116]. As result, its clinical utility remains in flux.

### Copy number variations

Gains or losses of areas of somatic DNA (i.e., NKX3.1) can have carcinogenic consequences, and PCa overall is characterized by loss of genomic material [117]. Specific gains or deletions as well as the overall burden of copy number variations (CNVs) may have a prognostic role in PCa pathogenesis.

Tsuchiya *et al*. investigated specific chromosome 8 abnormalities, and loss of 8p22 was associated with an increased risk of BCR and metastatic progression [118]. Loss of 8p22 in prostate biopsy specimens was also associated with PCa radioresistance after image-guided XRT with increased positive biopsies posttreatment [119]. Liu *et al*. studied the 20 most significant CNVs (15 deletions, five amplifications) in two RP cohorts and found that gain of *MYC* and deletion of *PTEN* were significantly associated with PCa-related death (after controlling for pathological stage, GS and initial PSA level) [120]. CNVs of *MYC* and *PTEN* were similarly found to be prognostic factors for disease relapse in PCa patients undergoing XRT (HR: 2.58) [121].

Paris *et al*. found that specific DNA-based biomarkers were associated with advanced disease stage (loss at 8p23.2) and were found to be predictive of postoperative recurrence (gain at 11q13.1) independent of tumor stage and grade [122]. The authors also suggest that a combined set of 39 loci termed the Genomic Evaluators of Metastatic Prostate Cancer (GEMCaP) is associated with PCa recurrence and metastasis. Subsequently, the GEMCaP was demonstrated to offer additional prognostic information above the Kattan nomogram for disease recurrence in high-risk node-negative PCa cases after RP (accuracy = 78% of nomogram + GEMCaP vs 65% of nomogram alone) [123].

As noted from the above studies, CNV analysis may have a prognostic role in PCa patients, but standardization of methods and additional validations studies are required before clinical applications can be planned.

### *PTEN*

One of the most frequently deleted genes in human cancer is a *PTEN* deletion on chromosome 10, which dephosphorylates lipid-signaling intermediates resulting in deactivation of PI3K signaling, thus controlling cell proliferation and growth [124]. Saal *et al*. initially correlated *PTEN* loss with poor outcomes in a variety of malignancies [125]. The prognostic value of *PTEN* deletion in PCa has subsequently been investigated in several studies. Leinonen *et al*. demonstrated a higher frequency of *PTEN* loss in more advanced cases (castrate-resistant PCa [CRPC] compared with localized disease in RP cases) and that *PTEN* loss was associated with shorter progression-free survival (PFS) in ERG-positive tumors [126]. Yoshimoto *et al*. reported that homozygous *PTEN* deletion in prostate specimens was independently associated with BCR risk after RP and was clearly associated with *TMPRSS2*: *ERG* gene expression [127]. Furthermore, *PTEN* loss at the time of RP correlated with clinical parameters of more advanced disease, such as extraprostatic extension and seminal vesicle invasion [128]. Krohn *et al*. examined over 4700 RP specimens and 57 CRPC cases, and the authors concluded that *PTEN* loss was independently associated with adverse clinicopathological features and was predictive of BCR and worse PFS after surgery [129]. ERG status, however, did not affect the predictive value of *PTEN* loss. Reid *et al*. showed that *PTEN* loss without *TMPRSS2*: *ERG* gene fusion on FISH and IHC was associated with worse CSS in castrate-sensitive cases. This is contrast to prior studies linking *PTEN* loss and positive *TMPRSS2*: *ERG* gene expression with worse outcomes [130]. Finally, McCall *et al*. compared *PTEN* status in both castration-sensitive and CRPC, noting that *PTEN* loss was independently associated with worse CSS but only in castrate-sensitive cases [131]. *PTEN*-negative tumors, however, have been associated with shorter survival in CRPC in the postchemotherapy setting during treatment with abiraterone [132].

Other markers have been tested in combination with *PTEN* loss for prognostic information including tumor protein *p27* gene loss, *HO-1* overexpression and *HER2/3* overexpression. Halvorsen *et al*. reported that combined loss of *PTEN* and *p27* expression defined a group of tumors based on RP specimens associated with increased tumor diameter, seminal vesicle invasion, increased pathological stage and elevated tumor cell proliferation detected by antigen KI-67 [133]. Loss of *PTEN* and *p27* expression was an independent predictor of time to BCR and clinical recurrence. Li *et al*. assessed the occurrence of both *HO-1* expression and *PTEN* deletion in men with localized and CRPC [134]. HO-1 epithelial expression was statistically different between benign prostate tissue, high-grade PIN, localized PCa and CRPC (p < 0.001). Although neither *HO-1* overexpression nor *PTEN* deletion alone in localized PCa was independently associated with PSA relapse, the combined status of both markers correlated with disease progression (p = 0.01). Inhibition of *HO-1* also strongly reduced cell growth and invasion *in vitro* and inhibited tumor growth and lung metastasis formation in mouse models. Finally, Ahmad *et al*. showed that patients who developed prostate tumors with low levels of *PTEN* and high levels of *HER2/3* have a poor prognosis [135]. These tumors, however, responded favorably to MAPK enzyme (MEK) inhibition by restoring the *PTEN* loss.

Loss of the *PTEN* gene and protein also shows promise as a prognostic biomarker in PCa patients on AS. *PTEN* status on RP specimens assessed by FISH has shown to be an independent predictor for preoperative PSA and GS with *PTEN* homozygous deletion associated with worse disease stage [136]. Tumors with *PTEN* protein loss were also more likely to be upgraded at the time of RP than those without loss, even after adjusting for age, preoperative PSA, clinical stage and race (OR: 3.04; p = 0.035) [137]. *PTEN* loss in Gleason 6 biopsy specimens identified a unique subset of prostate tumors at increased risk of upgrading at the time of RP. These data provide evidence that the assessment of *PTEN* status can improve Gleason accuracy and highlight a path toward the clinical use of molecular markers to augment pathologic grading.

### Peripheral blood & circulating tumor cells

In addition to genetic information, peripheral blood is a potential source for genomic tumor characterization using free circulating nucleic acids, whole blood transcripts or circulating tumor cells (CTCs). Bastian *et al*. reported an increasing quantity of circulating cell-free DNA independently associated with the risk of BCR after RP [138]. Ross *et al*. examined a six-gene panel (consisting of *ABL2, SEMA4D, ITGAL, C1QA, TIMP1* and *CDKN1A*) in CRPC patients with significantly improved prognostic value compared with a clinical model alone (AUC = 0.90 vs 0.65; p = 0.0067) [139], and Olmos *et al*. identified a nine-gene signature analyzed from blood messenger RNA (mRNA) in CRPC patients that was independently associated with worse OS [140]. Specific circulating miRNAs found not only in tumor tissue but also in the plasma of PCa patients, such as miRNA-375 and miRNA-141, have also been shown to be associated with advanced disease [141].

Danila *et al*. investigated the expression of five genes frequently detected in PCa cells (*KLK3, KLK2, HOXB13, GRHL2* and *FOXA1*) from whole blood transcripts as well as detection of CTCs [142]. Both an unfavorable CTC count (five or more cells) and detection of two or more gene transcripts had similar significant prognostic value for the risk of PCa-related death, and when combined, additional prognostic value was demonstrated. Additionally, KLK3, PCA3 and TMPRSS2:ERG mRNA could be detected in the peripheral blood of CRPC patients but not in healthy controls [143]. Decreased expression levels of these genes, however, were noted after docetaxel treatment, suggesting a potential role for treatment monitoring. Finally, Scher *et al*. demonstrated that a biomarker panel using CTC count and lactate dehydrogenase (LDH) was a surrogate for survival at the individual patient level in the Phase III trial of abiraterone plus prednisone versus prednisone alone (COU-AA-301) for patients with CRPC [144]. We suspect that peripheral blood markers and CTCs will continue to gain traction for use in future clinical studies as they can be easily collected with minimal patient morbidity.

## Genetic panels in PCa prognosis

Multiple genetic markers are often necessary to provide enough prognostic information for clinical decision-making versus any single genetic abnormality alone. Panels evaluating the differential expression of multiple genes are ideally created using knowledge of well-known carcinogenic pathways in PCa [145]. There is a risk of chance associations due to the number of genetic mutations associated with prostate malignancy, but blinded marker analysis and external validation is essential before any clinical application can be considered [146]. Additionally, the biomarkers in the panel have to provide additional independent predictive ability above and beyond standard clinical and pathological characteristics. Recommendations for reporting the clinical utility of biomarkers in oncology are, therefore, essential to follow [147].

Three commercially available RNA-based genetic panels that have been externally validated in the PCa population and are currently utilized in clinical practice are listed in Table 3. It is important to note that there is virtually no overlap between these panels even though they include a total of 85 genes. Additionally, there are no comparative or prospective studies evaluating these expression panels head-to-head in the same patient cohort. The commercial assays may also be less helpful because of the difficulties of using them effectively on the variable amount of tumor present at first diagnosis in needle cores.

Lalonde *et al*. also recently reported on a panel of DNA-based indices in 126 low-to-intermediate-risk PCa patients in order to develop four genomic subtypes for PCa that were prognostic for relapse after both XRT (HR: 4.5; AUC = 0.70) and RP (HR: 4.0; AUC = 0.57) and whose effect was magnified by intratumoral hypoxia (HR: 3.8; AUC = 0.67) [150]. This novel 100-loci DNA signature was subsequently externally validated in two independent cohorts of RP specimens from low-risk to high-risk patients, and it accurately classified treatment outcomes and identified patients who were most likely to fail treatment within 18 months (HR: 2.9; AUC = 0.68; p = 0.004).

### Prolaris^®^

Cuzick *et al*. identified a 46-gene-expression model (31 cell-cycle progression genes and 15 housekeeping genes) via quantitative PCR on RNA extracted from PCa tumor samples [145]. This cell cycle progression (CCP) score was evaluated retrospectively in a cohort of RP and TURP patients managed conservatively. The primary end point was BCR in the RP group and OS in the TURP group. The CCP score was an independent predictor of BCR for RP patients on univariate (HR: 1.89) and multivariate (HR: 1.77) analysis, and it was also strongly associated with time to death from PCa for TURP patients on univariate (HR: 2.92) and multivariate (HR: 2.57) analysis. CCP was also found to be a stronger prognostic factor than any other measured variable including PSA.

This gene panel (commercially available as the Prolaris test [Myriad Genetics, UT, USA]) was subsequently externally validated using biopsy and TURP specimens in 349 patients managed conservatively with the primary end point being PCa-specific mortality [151]. The CCP score was again independently associated with PCa-related death on univariate (HR: 2.02) and multivariate (HR: 1.65) analysis with GS and PSA providing significant additional contributions.

The Prolaris test has also been externally validated in RP studies. Cooperberg *et al*. analyzed 413 men who underwent RP and determined whether the CCP score (analyzed on RP tissue) could predict BCR (defined as two PSA levels ≥0.2 ng/ml) [148]. The CCP score was also assessed for independent prognostic utility beyond a stand postoperative risk assessment instrument such as the Cancer of the Prostate Risk Assessment post-Surgical (CAPRA-S) score, which uses pathological data from RP to predict PCa recurrence and mortality [152] and has been externally validated in over 2500 men across multiple institutions (concordance index [c-index] = 0.72 for BCR and 0.85 for PCa-specific mortality) [149]. CCP was independently predictive of BCR after RP on univariate (HR: 2.1) and multivariate (HR: 1.7) analysis after adjusting for the CAPRA-S score. CCP also was able to substratify patients with low clinical risk as defined by CAPRA-S ≤2 (HR: 2.3). Combining the CCP and CAPRA-S scores improved the c-index for both the overall cohort and the low-risk subset, suggesting that the combined score outperformed both individual scores in clinical decision-making (c-index = 0.77 for genetic + clinical model vs 0.73 for clinical model alone).

Bishoff *et al*. also evaluated the CCP score as a predictor of BCR and metastatic disease based on pre-RP biopsy tissue in 582 men treated with RP for clinical localized PCa [153]. CCP was the strongest predictor of BCR on univariate (HR: 1.6) and multivariate (HR: 1.47) analysis, and it was also the strongest predictor of metastatic disease on univariate (HR: 5.35) and multivariate (HR: 4.19) analysis after adjusting for other clinical variables.

Freedland *et al*. tested the prognostic utility of the Prolaris test in 141 PCa patients treated with external beam radiation therapy (EBRT) as their primary curative therapy using pretreatment diagnostic prostate biopsy specimens to predict BCR. BCR was defined using the Pheonix definition, and half of the cohort was African–American. The BCR rate was 13% (n = 19), and the CCP score significantly predicted BCR on univariate (p = 0.0017) and multivariate (p = 0.034) analysis accounting for GS, PSA, percent-positive cores and use of ADT. The CCP score also was associated with PCa-specific mortality at 10 years (p = 0.013).

The impact of the CCP score was prospectively analyzed based on physician-completed surveys regarding PCa treatment recommendations in 305 men before and after they received the Prolaris test results [154]. Clinicians also rated the influence of the test on treatment decisions. Surprisingly, the Prolaris test changed physician treatment recommendations in 65% of cases, and in 40% there was a reduction in treatment burden (interventional treatment changed to noninterventional). The long-term impact of these changes on patient outcomes, however, is not known.

### Oncotype DX^®^

Klein *et al*. identified 17 genes through PCR based on 441 RP specimens from low- to intermediate-risk patients representing multiple biological pathways in PCa out of 732 candidate genes (288 predicted clinical recurrence and tumor multifocality, and 198 were predictive of aggressive disease after adjustment for PSA, GS and clinical stage) [155]. This 17-gene-expression panel was further confirmed in 167 pre-RP biopsy specimens to create a multigene, expression-based signature called the Genomic Prostate Score (GPS). This algorithm was subsequently internally validated in 395 needle biopsies from contemporary patients who were candidates for AS to determine its ability to predict clinical recurrence, PCa-related death and adverse pathological features at the time of RP. In this validation study, the GPS predicted high-grade (Gleason ≥4 + 3; OR: 2.3) and high-stage (pT3 or higher; OR: 1.9) disease on RP specimens after controlling for established clinical factors (age, PSA, clinical stage, biopsy GS), and this was similarly true after inclusion of a clinical risk model (i.e., CAPRA-S; OR: 2.1).

This test (commercially available as the Oncotype DX test [Genomic Health, CA, USA]) was subsequently externally validated by Cullen *et al*. in 431 prostate biopsies from men with very low-, low- or intermediate-risk PCa based on National Comprehensive Cancer Network (NCCN) guidelines [156]. GPS scores were correlated with BCR, adverse pathology (primary Gleason patter 4 or any pattern 5, pT3 disease) and metastatic recurrence. GPS scores did not vary across race with similar distributions of results, and it was an independent predictor of BCR (HR: 2.7), time to metastases (HR: 3.8) and adverse pathology (OR: 3.3) after adjusting for the NCCN risk group. The Oncotype DX test performed on a patient's original prostate needle biopsy, therefore, may predict the aggressiveness of PCa and help men make decisions regarding immediate treatment versus AS.

### Decipher^®^

Erho *et al*. presented a case-control study that analyzed a 22-gene-expression signature in men with early clinical metastasis after rising PSA to predict survival after RP [157]. The 22-gene panel (based on the primary tumor in the RP specimen) was the only significant prognostic factor for early metastasis and PCa-specific death on multivariable analysis, and it had a good correlation with DSS on internal validation (AUC = 0.75). Patients with higher scores experienced earlier death from PCa and reduced OS, suggesting its use as an identifier of aggressive prostatic malignancy.

This 22-gene panel (commercially available as the Decipher test [GenomeDX Biosciences, BC, USA]) was externally validated in multiple studies. Cooperberg *et al*. compared the CAPRA-S score and the Decipher genomic classifier (GC) as a predictor of PCa-specific mortality in 185 men at high risk of recurrence (PSA >20, GS ≥8, stage pT3b) after RP of whom 25 experienced PCa-associated death [158]. The c-indices for CAPRA-S and Decipher GC were 0.75 and 0.78, respectively, and Decipher GC reclassified many men stratified to high-risk based on the CAPRA-S score ≥6. Both high CAPRA-S and Decipher GC scores were independently predictive of PCa-specific mortality (HR: 2.36 and 11.26, respectively) with a cumulative incidence of PCa-related death of 45% at 10 years. This suggests that integration of genomic and clinical classifiers may enable better identification of post-RP patients at high-risk for death who should be considered for more aggressive secondary therapies and clinical trials.

Karnes *et al*. also evaluated 219 men at high risk of recurrence (PSA >20, GS ≥8, stage pT3b) with the Decipher test based on the genomic information from the primary tumor in the RP specimen [159]. The Decipher GC AUC was 0.79 for predicting 5-year metastasis after RP, which exceeded that of clinical models. The Decipher GC was also the predominant predictor of metastasis 5 years after RP on multivariate analysis with a cumulative incidence of 2.4, 6.0 and 22.5% in patients with low (60%), intermediate (21%) and high (19%) scores, respectively (p < 0.001).

In another study, 85 clinically high-risk RP patients who developed BCR after surgery were analyzed using the Decipher GC as a predictor of metastatic disease progression [160]. The AUC of Decipher GC for predicting metastasis after BCR was 0.82 compared with 0.64 for GS, 0.69 for PSADT and 0.52 for time to BCR. It was the only variable associated with metastatic progression on multivariate analysis (p = 0.003), and it performed superiorly to models solely based on clinicopathological features (i.e., CAPRA-S).

Finally, studies have also evaluated the utility of Decipher GC to predict which men would benefit from adjuvant XRT therapy as well as to forecast outcomes after postoperative XRT following RP. Den *et al*. calculated Decipher GC scores from the primary tumor specimens of 188 patients with pT3 or margin-positive PCa after RP who received post-RP XRT [161]. The primary end point was clinical metastasis, and the prognostic accuracy of Decipher GC was compared with other routine clinical and pathological features. Decipher GC and pre-RP PSA were both independent predictors of metastasis (p < 0.01) with a cumulative incidence at 5 years of 0, 9 and 29% for low, average and high GC scores. For patients with low GC scores, there was no difference in the cumulative incidence of metastasis comparing patients who received adjuvant vs salvage XRT (p = 0.79), but for patients with high GC scores, the cumulative incidence of metastasis at 5 years was significantly lower in patients who received adjuvant vs salvage XRT (6 vs 23%; p < 0.01). These findings suggest that patients with high Decipher GC scores may benefit from adjuvant XRT, while patients with low Decipher GC scores are best treated with salvage XRT.

This same group also reported on the ability of the Decipher GC score to predict BCR and distant metastasis in men receiving XRT after RP [162]. The authors evaluated 139 PCa patients with pT3 or positive margins after RP who received postoperative XRT (both adjuvant and salvage) and found that the addition of Decipher GC improved the AUC of a clinical model (i.e., Stephenson model) from 0.70 to 0.78 and from 0.70 to 0.80 as a predictor of BCR and distant metastasis, respectively. On multivariate analysis, high Decipher GC scores were independent predictors of BCR (HR: 8.1) and distant metastasis (HR: 14.3) with a cumulative incidence of 21, 48 and 81% for BCR and 0, 12 and 17% for distant metastasis for low, intermediate and high GC scores, respectively. The Decipher GC, therefore, may be predictive of oncological outcomes after post-RP XRT and improve decision-making in high-risk cases.

## Biomarkers in PCa response to treatment

Genetic markers that can predict response or resistance to treatment could be valuable in the selection of a therapy with the greatest likelihood of efficacy in an individual patient. They may also categorize patients into clinical trials based on the presumed likelihood of response to the tested agents.

It is well known that ADT is first-line therapy for metastatic PCa. Inherited genetic factors, however, may account for variability in response to ADT. Ross *et al*. identified three polymorphisms (*CYP19A1, HSD3B1* and *HSD17B4*) independently associated with longer time to progression (TTP) during ADT on multivariate analysis [163]. Patients carrying more than one of these polymorphisms demonstrated a better response to ADT than patients carrying none (p < 0.0001). The *SLCO1B3* polymorphism, on the other hand, was found to be associated with a shorter time to androgen independence in PCa patients due to its enhanced effect on testosterone import [164]. Yang *et al*. also identified three SNPs in *SLCO2B1* associated with shorter TTP on ADT. Individuals carrying the *SLCO1B3* and *SLCO2B1* genotype allowed for more efficient import of androgen and enhanced cell growth associated with a median 2-year worse TTP on ADT [165].

Although ADT remains first-line treatment for metastatic PCa, predicting the response of CRPC to various novel agents may have added value. Theoretically, tumors with AR amplification or overexpression would be expected to respond well to abiraterone, which further decreases circulating androgens. Conversely, tumors with AR mutations leading to promiscuity to nonandrogen substrates or loss of AR expression would be expected to respond well to enzalutamide, which prevents AR nuclear import. CRPC mediated through active splice variants lacking the ligand-binding domain at the carboxy-terminal of the AR protein might require novel agents targeting the amino-terminal of the protein [166]. Multiple markers have also been implicated in taxane resistance in CRPC, including elevated class III β-tubulin expression [167,168]. These markers, however, have yet to be prospectively validated.

Peripheral blood genetic information may be useful to predict therapeutic response in CRPC. Pretherapy CTC counts have been demonstrated to predict response to therapy, and a decrease in the number of CTCs after therapy had a greater predictive value than the classic 50% PSA decrease [169]. This was observed after treatment with both docetaxel and abiraterone [170,171]. Antonarakis *et al*. also recently reported that a splice variant of the androgen receptor (AR-V7) could be detected in CTCs from CRPC patients [172]. AR-V7-positive patients had lower PSA response rates to abiraterone and enzalutamide than AR-V7-negative patients (0 vs 68% [p = 0.004] and 0 vs 53% [p = 0.004], respectively). They also had a shorter PSA PFS (median = 1.3 months vs not reached [p < 0.001] and 1.4 vs 6.0 months [p < 0.001], respectively), shorter clinical or radiographic PFS (median = 2.3 months vs not reached [p < 0.001] and 2.1 vs 6.1 months [p < 0.001], respectively) and shorter OS (median = 10.6 months vs not reached [p = 0.006] and 5.5 months vs not reached [p = 0.004], respectively). One limitation of this study, however, is the fact that AR-V7-positive patients had a greater overall disease burden than AR-V7-negative patients, so it is unclear whether AR-V7 represents a predictive factor of response to abiraterone and enzalutamide in CRPC or simply a marker of more advanced disease.

## Conclusion

Newer molecular biomarkers allow the clinician to better evaluate patients for the detection of clinically significant prostate cancer preventing over diagnosis and overtreatment. They are becoming a powerful tool for risk-stratification and for directing treatment.

## Future perspective

A possible future algorithm in the diagnosis and prognosis of PCa is demonstrated in Figure 1. Challenges still remain, however, in the development of genetic markers in the PCa population. PCa is a heterogeneous disease both within a single tumor locus (intrafocal heterogeneity) and between different tumor deposits (interfocal heterogeneity) [173]. Ongoing abnormal mutational processes give rise to extensive branching evolution and cancer clone mixing, which can be demonstrated by the coexistence of multiple cancer lineages harboring distinct ERG fusions within a single PCa nodule [174]. There is a field effect of molecular changes as benign areas of the same prostate can show cancer-related genetic changes [175]. Discordance may also exist between metastatic versus primary lesions, so tissue sampling is of the utmost importance. The morbidity and costs associated with biopsy of metastatic or primary lesions, however, may dissuade patients from participation in clinical trials. CTCs in the peripheral blood may offer an alternative option for disease monitoring since genetic and molecular changes in prostate CTCs have shown to predict response to therapy in metastatic lesions.

Second, although combinations of multiple markers (gene-expression profiles) have been tested as predictors of disease prognosis and clinical response in PCa with three commercially approved assays available, there seems to be no commonality in the prognostic genes identified, raising questions as to the randomness of these identified biomarkers and whether they just represent downstream pathways in carcinogenesis rather than inciting genetic mutations. These genetic panels also have to be validated in future prospective studies to truly gain credibility, and testing them head-to-head with known clinicopathological risk factors will ultimately determine their added prognostic value. Inflammatory biomarker panels may provide an alternative option in the near future as emerging data suggest that an improved understanding of the tumor–host microenvironment within the prostate will lead to new immunobiomarkers that may be very informative.

Finally, although large-scale efforts have been placed into identifying genetic markers and molecular indicators in the diagnosis and prognosis of PCa, investigation into PCa prevention is still lacking. Despite knowledge of inherited alleles that contribute to PCa development, genetic manipulation of these susceptibility loci has still not been possible to reduce the incidence of disease and the burden of treatment in a high-risk population. Additionally, whether genetic or biochemical engineering can be used to change the natural history and the overall aggressiveness of PCa is still not known and may be the subject of future investigation.

**Table 1.** **Description of prostate-specific antigen-based markers utilized in clinical practice for prostate cancer diagnosis.**

**Marker**	**Description**
Age-adjusted PSA	Age-specific reference limits for serum total PSA stratified by race (i.e., Caucasian, African–American)
PSA doubling time	Time in months or years for the total PSA to double
PSA velocity	The rate of change of PSA measured in ng/ml per year (change in PSA over time)
Free PSA	Serum PSA unbound to alpha-1-anti-chymotrypsin
Percent-free PSA	Ratio of free PSA to total PSA
ProPSA	The precursor of PSA, which is an inactive 224-amino acid protein secreted by prostatic cells
PSA density	Total PSA divided by PSA volume (length × width × height × π/6) in ml or cc

PSA: Prostate-specific antigen.

**Table 2.** **Commercially available biomarkers utilized in clinical practice for prostate cancer diagnosis.**

**Panel**	**Specimen**	**Description**	**AUC for PCa detection**	**Sensitivity (%)**	**Specificity (%)**	**Ref.**
PCA3	Urine	Measures noncoding RNA only expressed in human prostate tissue	0.658	66	76	[34]
PHI	Serum	Combines total, free and (-2)proPSA	0.703	80	45	[35]
4Kscore^®^	Serum	Consists of total PSA, free PSA, intact PSA and KLK2	0.711	–	–	[36]
ConfirmMDx^®^	Prostate biopsy tissue	Measures the degree of methylation of GSTP1, APC and RASSF1	–	68	64	[37]

AUC: Area under curve; PCa: Prostate cancer; PHI: Prostate health index; PSA: Prostate-specific antigen.

**Table 3.** **Commercially available RNA-based gene panels utilized in clinical practice for prostate cancer prognosis.**

**Panel**	**Company**	**Tissue type**	**Number of genes**	**End point**	**Ref.**
Prolaris^®^	Myriad Genetics	Radical prostatectomy, prostate biopsy	31	BCR, DSS, MFS	[140]
Oncotype DX^®^	Genomic Health Inc.	Radical prostatectomy, prostate biopsy	17	BCR, adverse RP pathology, MFS	[148]
Decipher^®^	GenomeDX Biosciences	Radical prostatectomy	22	BCR, DSS, MFS	[149]

BCR: Biochemical recurrence; DSS: Disease-specific survival; MFS: Metastasis-free survival; RP: Radical prostatectomy.

**Executive summary**

Advances in the genotyping and understanding of biochemical pathways involved in prostate cancer (PCa) development and carcinogenesis have led to the possibility of personalized genetic profiling of primary and metastatic tumor cells.
These biomolecular signatures may become readily available for routine clinical decision-making in PCa patients ranging from the need for repeat biopsy, initial treatment selection, decisions about adjuvant therapy or selection of treatments for advanced disease.
Large clinical trials involving multi-institutional collaborations will be necessary to prospectively validate the utility of these genetic biomarkers in the diagnosis and treatment of PCa.
Future research should also focus on biogenetic engineering in PCa prevention as well as targeted agents to minimize disease aggressiveness in order to reduce the incidence and burden of this malignancy.
